# Review of calibration strategies for discrete element model in quasi-static elastic deformation

**DOI:** 10.1038/s41598-023-39446-2

**Published:** 2023-08-15

**Authors:** Xianyang Liu, Qunwei Wang, Yongwei Wang, Qinxi Dong

**Affiliations:** 1 School of Civil Engineering and Architecture, Hainan University, Haikou, 570228 China; 2 China National Chemical Engineering NO. 13 Construction CO., LTD, Cangzhou, 061000 China; 3 Key Laboratory of Equipment Safety and Intelligent Technology for Guangzhou Rail Transit System, Guangzhou, 510430 China

**Keywords:** Engineering, Civil engineering

## Abstract

This study first reviewed theories of the mechanical response of structures under loading, and the discrete element method provides a route for studying mechanical response including elastic deformation and structure failure. However, the direct acquisition of the microscopic parameters from the governing equations of the discrete element method via experiments encounters challenges. One possible strategy to obtain these microscopic parameters is parameter calibration that are widely used by researchers. Secondly, the governing equations and failure criterion of the discrete element method are summarized, and the microscopic parameters that would be calibrated are pinpointed. Next, the principles of classical calibration methods of discrete element method are explicated in detail, alongside the validation and discussion of their properties. Lastly, this study examined the applicability of calibrated parameters and points out that the size ratio, porosity, maximum radius, and minimum radius of particles should be identical in both the geometric calibration model and that for applications.

## Introduction

When an external force is applied to a structural system, mechanical responses occur. Classical continuum mechanics is commonly employed in the investigation of these mechanical responses, with the governing equations involving partial differential equations. However, when classical continuum mechanics encounters fractures, it is faced with difficulties due to the nonexistence of derivatives at discontinuities^1^ (e.g., fracture ).

Various methods have been proposed by researchers to address fracture-related issues, including phase field theory^2^, extended finite element method^3,4^, peridynamics^1^, and discrete element method^5^. Phase field theory for fractures utilizes a continuous damage function to approximate the presence of free discontinuity surfaces^6,7^. However, it should be noted that phase field fracture technology solely describes the progression of highly localized damage, and not the nucleation and propagation of discontinuities. Therefore, it is fundamentally a continuous field-based technology. The extended finite element method (XFEM) is a numerical method that adds a function capable of reflecting discontinuities to the displacement function of the traditional finite element method, The method utilizes the level set method to dynamically track interface changes, allowing for the resolution of various types of discontinuities, such as cracks, holes, and inclusions^8^. However, XFEM may encounter challenges when dealing with crack branching. Peridynamics, instead of relying on traditional differential equations, employs integral equations to avoid singularity at crack tips^1^. Peridynamics holds tremendous advantages in solving non-continuous problems such as fracture^9,10^. However, stiffness reduction issues around material boundaries may arise in peridynamics. The DEM regards materials as discrete media, where each block or particle moves according to Newton’s second law^5^. They can simulate displacement, rotation, sliding, and even separation. DEM can realistically and intuitively simulate fracture and other large deformation phenomena. The fracture of bulk systems comprised of particles initiates from the separation of particles. The disappearance of the force between two particles means the onset of a crack. With decades of development, DEM has been widely applied in various fields such as geotechnical engineering^11–16^, mining^17–20^ and agriculture^21–25^. Accordingly, several DEM software packages have been developed^26–29^.

Before conducting simulations using DEM, it is essential to determine the material parameters involved in the model. In classical continuum mechanics, material parameters such as Young’s modulus and Poisson’s ratio can be determined through experiments. However, parameters from DEM need to be specified at the microscopic level, such as normal contact stiffness and tangential contact stiffness which are called microscopic parameters. These microscopic parameters are different from the macroscopic parameters. It has difficulties to measure experimentally^30^. At present, the method for determining microscopic parameters in DEM is parameter calibration. It is noteworthy that this study focuses on the elastic deformation of the solid structure that is assumed to be composed of millions of particles under quasi-static loading. The study of elastic deformation primarily relies on the principles of elasticity theory while the dynamic particulate systems rely on other mechanics (e.g. theoretical mechanics)^31–38^. As a result, the key parameters and calibration methods differ significantly between the elastic structure and dynamic particulate systems. For instance, particle density is measured using a gas pycnometer, and the sliding friction coefficient is determined through the sliding friction test in dynamic particulate systems^31,32^. Linearly elastic deformation employs constitutive equations of linear elasticity. The fundamental macroscopic parameters in linear elasticity are Young’s modulus and Poisson’s ratio. These macroscopic parameters have a great influence on the deformation of structure^39^. In the context of linear elasticity, these macroscopic parameters correspond to the microscopic parameters of the discrete element model, namely the effective modulus and stiffness ratio.

With the widespread application of the discrete element method, scholars developed several calibration strategies. Yoon stated that the trial-and-error method was the only method available before 2006^40^. With the increasing application of DEM, the empirical method was proposed and the experience could be used to determine the initial values of the calibration. In 2007, the design of experiment (DOE) methodology was proposed for calibrating macroscopic and microscopic parameters through the utilization of experimental designs^40^. Machine learning has a remarkable capability in handling nonlinear issues. In 2011, the application of neural network training was utilized in DEM calibration^41^. Now various machine learning methods have been explored for parameter calibration^42–44^. In 2019, Qu made a contribution by presenting a theoretical derivation for DEM calibration^45^. The basic idea of this method is that the elastic energy density characterized by the DEM and that described by classical continuum mechanics are equivalent. In 2021, the evolutionary algorithms (e.g., particle swarm optimization and the differential evolution algorithm) were proposed for calibration without training data^46,47^. These calibration strategies were described and discussed in the following sections.

The primary goal of this paper is to review these calibration methods. The remainder of the paper is organized as follows: in “Basic concept of discrete element model” section describes the basic concepts of the discrete element method, including governing equations, failure criterion, and related microscopic parameters that should be calibrated; In “Review of calibration strategies” section introduces the principles and processes of the calibration methods and discusses their advantages and disadvantages; In “The applicability of calibrated parameters” section studies the applicability of calibrated parameter via particle distribution and particle radius; In “Conclusions” section presents recommendations and conclusions on the calibration methods based on the previous research.

## Basic concept of discrete element model

The DEM is proposed by cundall^5^ in the 1970s. The core of DEM is Newton’s second law. There are several constitutive models in DEM. The classification of constitutive models is usually divided into two categories: elastic and inelastic models. The elastic models can be divided into linear and nonlinear models. Hertz-Mindlin model and Johnson-Kendall-Roberts model belong to nonlinear models^48–50^. The linear contact model, linear contact bond model, and linear parallel bond model are linear models that were discussed in this study. This section provides a brief overview of the governing equations of DEM and the microscopic parameters involved.

### Governing equations

DEM has a strong capacity to simulate elastic deformation, crack initiation, crack evolution, and structural failure due to its ability to imitate the evolution of material crack formation^51–53^. This ability mainly relies on the governing equations of DEM^5^, which are as follows:

- Equilibrium equations:
  1 $$\begin{aligned} \sum _{j=1}^{N}\textbf{F} _{i,j}+\textbf{b}_{i}=m_{i}\textbf{a} _{i} \end{aligned}$$
- Constitutive equations:
  2 $$\begin{aligned} \left\{ {\begin{array}{*{20}{l}} \textbf{F} _{i,j}^{n}=K_{i,j}^{n}U_{i,j} ^{n}\textbf{n}\;\;\;\;\;\;\;\;\; \quad for \ U_{i,j} ^{n}> 0 \\ \bigtriangleup {\textbf{F}}_{i,j}^{s} =-K_{i,j}^{s}\bigtriangleup {U}_{i,j} ^{s} \textbf{s } \;\; for\;\left| F_{i,j}^{s} \right| < \left| \mu F_{i,j}^{n} \right| \end{array}} \right. \ \end{aligned}$$
- Kinematic admissibility and compatibility equations:
  3 $$\begin{aligned} \left\{ {\begin{array}{*{20}{l}} U_{i,j} ^{n}=r_{i}+r_{j}-\left| \textbf{x} _{i}-\textbf{x} _{j} \right| \\ \bigtriangleup U_{i,j}^{s}=V^{s} \bigtriangleup t \end{array}} \right. \ \end{aligned}$$

**Figure 1:**
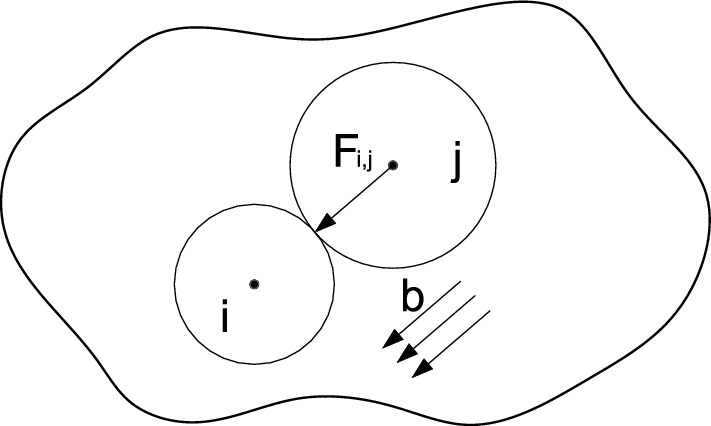
Interaction between particles.

In equilibrium equations, $\textbf{F} _{i,j}$ is the contact force exerted by particle j on particle i. N is the total number of particles in contact with particle i. $\textbf{b}_{i}$ is the body force acting on the particle i. $m_{i}$ is the mass of particle i. $\textbf{a}_{i}$ is the acceleration of particle i. The interaction between the above particles is shown in Fig. 1.

And $\textbf{F} _{i,j} =\textbf{F} _{i,j}^{n} +\textbf{F} _{i,j}^{s}$, where $\textbf{F} _{i,j}^{n}$ is the decomposition of $\textbf{F} _{i,j}$ in the normal direction, and $\textbf{F} _{i,j}^{s}$ is the decomposition of $\textbf{F} _{i,j}$ in the tangential direction.

In constitutive equations, $K_{i,j}^{n}$ is the normal contact stiffness between particles i and j. $K_{i,j}^{s}$ is the tangential contact stiffness. $U_{i,j} ^{n}$ is the normal overlap. Note that the stiffness and overlap from the dynamic particulate systems are commonly described by *k* and $\delta$, respectively^54^. Because the linear model cannot bear tension between particles. Equation (2) exists when $U_{i,j} ^{n}$ is greater than 0. $\textbf{n}$ is the normal unit vector at the contact point of two particles i, j. $\bigtriangleup {\textbf{F}}_{i,j}^{s}$ is the increment of tangential contact force between particles i, j. $\bigtriangleup {U}_{i,j}^{s}$ is the tangential increment of overlap. $\textbf{s}$ is the tangential unit vector. $\mu$ is the friction coefficient between the particles, which determines whether frictional slip occurs.

In kinematic admissibility and compatibility equations, $r_{i}$ and $r_{j}$ are the radius of particles i, j. $\textbf{x} _{i}$ and $\textbf{x} _{j}$ are the coordinate vectors of particles i, j, respectively. $\bigtriangleup {U}_{i,j}^{s}$ is the relative displacement increment generated tangentially in time $\bigtriangleup t$. $V^{s}$ is the relative tangential velocity between contact particles.

**Figure 2:**
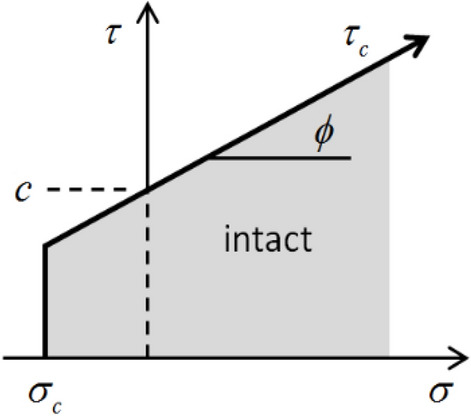
Failure criterion in linear parallel bond model^29^.

### Other models

Apart from the linear model, the bond model is another commonly utilized model in the field^55^. The major distinction between them is the fact that bond models permit the development of tensile forces, as evidenced by their disparate constitutive equations.

- Constitutive equation of linear contact bond model
  4 $$\begin{aligned} \textbf{F} _{i,j}^{n}=K_{i,j}^{n}U_{i,j} ^{n}\textbf{n} \end{aligned}$$
- Constitutive equations of the linear parallel bond model
  5 $$\begin{aligned} \left\{ {\begin{array}{*{20}{l}} {\bigtriangleup {\textbf{F} _{i,j}^{n} }} ={{k} _{i,j}^{n} } A\bigtriangleup {U}_{i,j} ^{n} \textbf{n}\\ {\bigtriangleup {\textbf{F} _{i,j}^{s} }} =-{{k} _{i,j}^{s} } A\bigtriangleup {U}_{i,j} ^{s} \textbf{s} \end{array}} \right. \ \end{aligned}$$

Where ${\bigtriangleup {\textbf{F} _{i,j}^{n} }}$ and ${\bigtriangleup {\textbf{F} _{i,j}^{s} }}$ are the normal and tangential contact force increments, respectively. ${{{k} _{i,j}^{n} } }$ is the normal stiffness per unit area. ${{k} _{i,j}^{s} }$ is the tangential stiffness per unit area. And A is the bonded cross-sectional area. $\bigtriangleup {U}_{i,j}^{n}$ is the normal relative displacement increment.

### Failure criterion

The failure of the bond is mainly controlled by the three parameters of tensile strength ${\sigma _{c} }$, cohesion *c* and internal friction angle ${\phi }$^29^. From Fig. 2, it is shown that the bond breaks when the tensile strength ${\sigma _{c} }$ or shear strength $\tau _{c}$ is reached. The shear strength is determined by ${\tau _{c} } ={c} +\sigma tan{\phi }$, where $\sigma ={\textbf{F} _{i,j}^{n}} /{{A} }$ is the average normal stress acting on the cross-section of the parallel bond. When ${\phi }$ is 0, $\tau _{c}$ becomes a constant, which also applies to the linear contact bond model.

### Microscopic parameters

From the above, it is shown that different models require different microscopic parameters. Among the models, the normal and tangential stiffness are related to the size of the particles. To facilitate the calibration, effective modulus $E^{*}= K_{i,j}^{n} l/2r_{min}$ where $l=r_{i}+r_{j}$, $r_{min}$ is the smallest radius between $r_{i}$ and $r_{j}$ and stiffness ratio $K^{*}=K_{i,j}^{n}/K_{i,j}^{s}$ are used. The specific microscopic parameters to be calibrated are listed in Table 1 below:

**Table 1:** Microscopic parameters to be calibrated.

	Linear model	Linear contact bond model	Linear parallel bond model
Emod $E^{*}$	Yes	Yes	Yes
Kratio $K^{*}$	Yes	Yes	Yes
Fric $\mu$	Yes	Yes	Yes
Shearf $\tau _{c}$		Yes	
Tenf $\sigma _{c}$		Yes	Yes
Pb_emod $\hbox {p}b\_E^{*}$			Yes
Pb_kratio $pb\_K^{*}$			Yes
Coh *c*			Yes
Fa $\phi$			Yes

(Pb_emod is bond effective modulus, Pb_kratio is bond normal-to-shear stiffness ratio).

**Figure 3:**
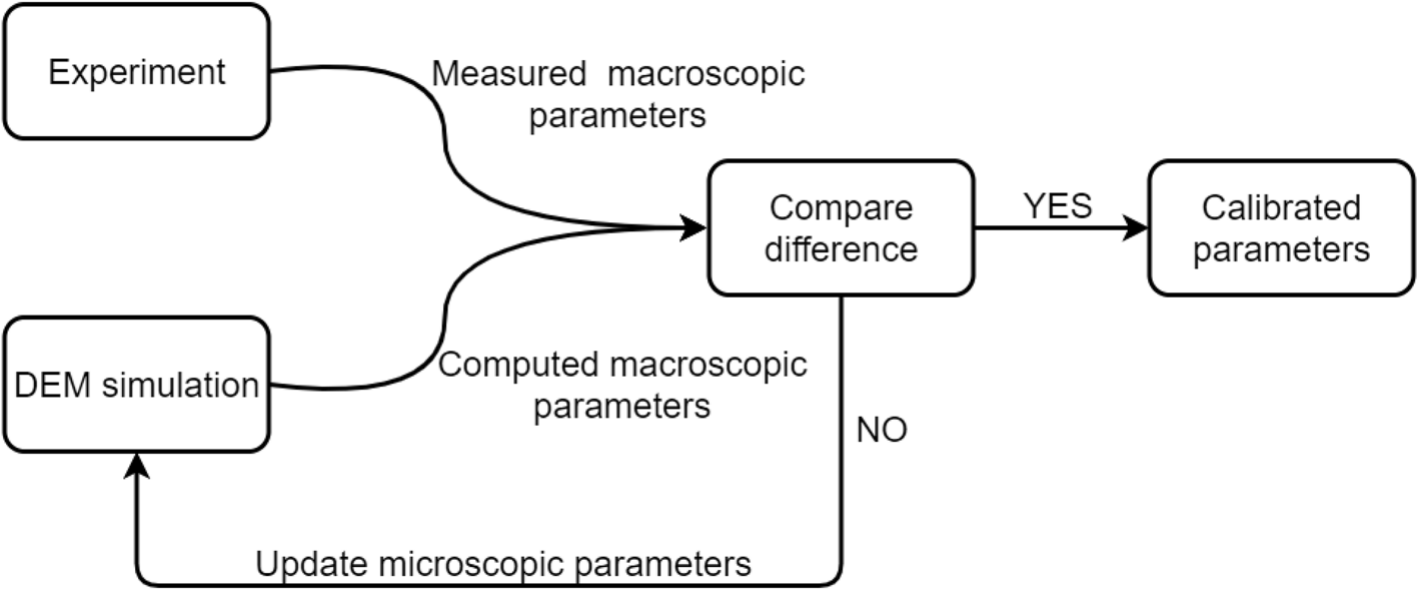
Trial and error method process.

## Review of calibration strategies

### Trial-and-error method

#### Calibration principle

Several researchers have conducted extensive studies on parameters calibration of DEM with the trial-and-error method^30,40,42,56^. Some researchers employed uniaxial testing in their research^57,58^. Figure 3 illustrates the calibration procedure of the trial-and-error method, which begins by obtaining the material’s macroscopic parameters through experiments. The DEM microscopic parameters are randomly selected for numerical simulation, and the resulting macroscopic parameters are calculated. If the error between the measured macroscopic parameters and the calculated macroscopic parameters does not meet the requirement, the DEM microscopic parameters are updated until the error meets the requirement. This process continues until the DEM-calibrated parameters are obtained.

Several researchers have conducted extensive studies on calibrating the parameters in DEM, with the trial-and-error method being a common method^30,40,42,56^. Figure 3 illustrates the calibration procedure of the trial-and-error method, which begins by obtaining the material’s macroscopic parameters through experiments. The DEM microscopic parameters are randomly selected for numerical simulation, and the resulting macroscopic parameters are calculated. If the error between the measured macroscopic parameters and the calculated macroscopic parameters does not meet the requirement, the DEM microscopic parameters are updated until the error meets the requirement. This process continues until the DEM-calibrated parameters are obtained.

For homogeneous, isotropic linear elastic materials, equation (6) or equation (7) is utilized to calculate the macroscopic parameters E and $\upsilon$ from simulation results of uniaxial compression.

6 $$\begin{aligned}{} & {} {\left\{ \begin{array}{ll} E=\frac{\bigtriangleup \sigma _{y} }{\bigtriangleup \varepsilon _{y} } \\ \quad \quad \quad \quad \quad for \ 3D \ and \ plane \ stress \\ \upsilon =-\frac{\bigtriangleup \varepsilon _{x} }{\bigtriangleup \varepsilon _{y} } \end{array}\right. } \end{aligned}$$

7 $$\begin{aligned}{} & {} {\left\{ \begin{array}{ll} {\upsilon }' =\frac{{\upsilon } }{1+{\upsilon } } \\ \quad \quad \quad \quad \quad \quad \quad plane \ strain \\ {E}' =E(1-({\upsilon }') ^{2} ) \end{array}\right. } \end{aligned}$$

Where $\bigtriangleup \sigma _{y}$ is the increment of component of the Cauchy stress tensor in the direction of the compression. $\bigtriangleup \varepsilon _{x}$ is the increment of the component of the infinitesimal strain tensor in the transverse direction. $\bigtriangleup \varepsilon _{y}$ is the increment of component of the infinitesimal strain tensor in the direction of the compression. And the stress component $\sigma _{y}$ can be calculated by equation (8).

8 $$\begin{aligned} \sigma _{y} =\frac{F}{A^{'} } \end{aligned}$$

Where F is the external force in the direction of uniaxial compression and $A^{'}$ is the cross-section area where F acts directly. The strain component $\varepsilon _{y}$ can be calculated by equation (9).

9 $$\begin{aligned} \varepsilon _{y} =\frac{L-L_{0} }{L} \end{aligned}$$

Where L is the original length of the specimen in the uniaxial compression direction. And $L_{0}$ is the change in length of the specimen in the uniaxial compression direction after deformation.

#### Validation

**Figure 4:**
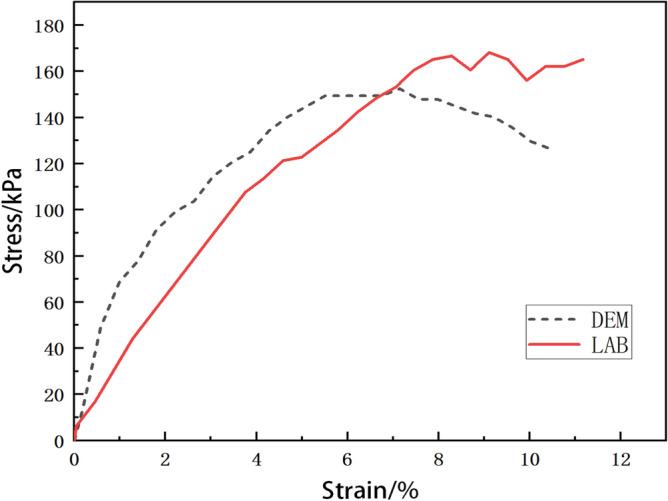
Comparison between experiment and simulation^59^.

After calibration, Zhou^59^ used the calibrated parameters to conduct simulations. By comparing the simulation and experimental results of Shanghai clay, as shown in Fig. 4, it was found that the curve obtained from the experiment did not fit the curve obtained from the simulation.

**Figure 5:**
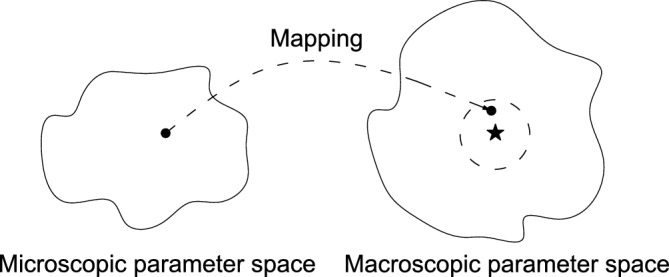
Calibration results and true solution.

The comparison between the stress-strain curves obtained from DEM simulation and experiments, as conducted by zhou^59^, is shown in Fig. 4. Figure 4 shows that the DEM simulated curve encounters a nonlinear deformation before reaching the peak. The experimental curve and the DEM simulated curve bifurcate largely at the beginning of loading. The strength from the experiment is 166.8 KPa while that of the DEM simulation is 149.5 KPa. The relative error between them is 10.3%. Note that the relative error is defined by $\left| strength \ from \ experiment- strength \ from \ DEM\right| /strength \ from \ experiment$. Moreover, the strain value of the peak point from the experimental curve is much larger than that of the DEM simulated curve. The DEM simulated data are not in good agreement with the test results. Because it has difficulties pinpointing the target of macroscopic parameters under the trial-and-error method (see Fig. 5). The star in the macroscopic parameter space on the right represents the true solution, and the dashed circle around it represents its neighborhood.

#### Discussion

The trial-and-error method is easy to operate. However, it has great difficulty to get satisfactory results due to a high degree of randomness. Meanwhile, it has high computational costs. Furthermore, the trial-and-error method lacks scientific rigor, and calibrated parameters may only map into a subset of the macroscopic parameter space close to the true solution, as shown in Fig. 5.

### Empirical method

Determining microscopic parameters to improve the agreement between simulation results and experimental data has been a longstanding challenge for researchers. With an increasing number of studies and applications of DEM, calibration techniques have been developed and refined over time, such as estimating appropriate initial values of certain material parameters based on prior knowledge of their expected ranges. Researchers^55,60–73^ have conducted DEM simulations of different materials, such as sand, clay, granite, and synthesized material-specific properties. With the widespread application of the discrete element method, scholars become more experienced in understanding material characteristics described by DEM. Zhang^74^ conducted triaxial simulations and observed that an increase in the normal bond strength led to large increments in the macroscopic elastic modulus and peak stress. Through sensitivity analysis, scholars have identified the sensitivities of macroscopic parameters to variations of the stiffness ratio^75^. Similarly, sensitivities of macroscopic parameters to the contact modulus were found following the order of the elastic modulus, the tensile strength, the compressive strength, and Poisson’s ratio. Zhou performed biaxial simulations on cohesive soils, revealing that the normal and tangential bond strength governed the material’s shear failure morphology^64^.

Not only the microscopic parameters but also the geometrical modeling influences the accuracy of the calibration. Some researchers^31,76–89^ have explored calibration strategies from the perspective of particle shape and extracted useful information, which can enhance the accuracy of the calibration process. Zhang conducted biaxial analyses using four distinct particle forms: circular, elongated (similar to rectangles), triangular-like, and square-like particles. Their findings indicated that, when the microscopic parameters were identical, the particle shape exerted a significant influence on the macroscopic characteristics of the particle samples ^76^. Similarly, Coetzee^77,90^ employed clusters composed of particles with various shapes. It was observed that clusters comprising eight or four particles demonstrated superior accuracy compared to those consisting of two clusters. Xu^91^ implemented digital imaging techniques to enable automated modeling for discrete element simulations.

The aforementioned studies represent the collective research experience of various scholars in the field of the discrete element method. These experiences can facilitate parameter calibration, such as estimating appropriate initial values of certain materials. This type of method is named the empirical method. This section provides an overview of the empirical method obtained from the two perspectives of particle shape and material parameters.

#### Calibration principle

**Figure 6:**
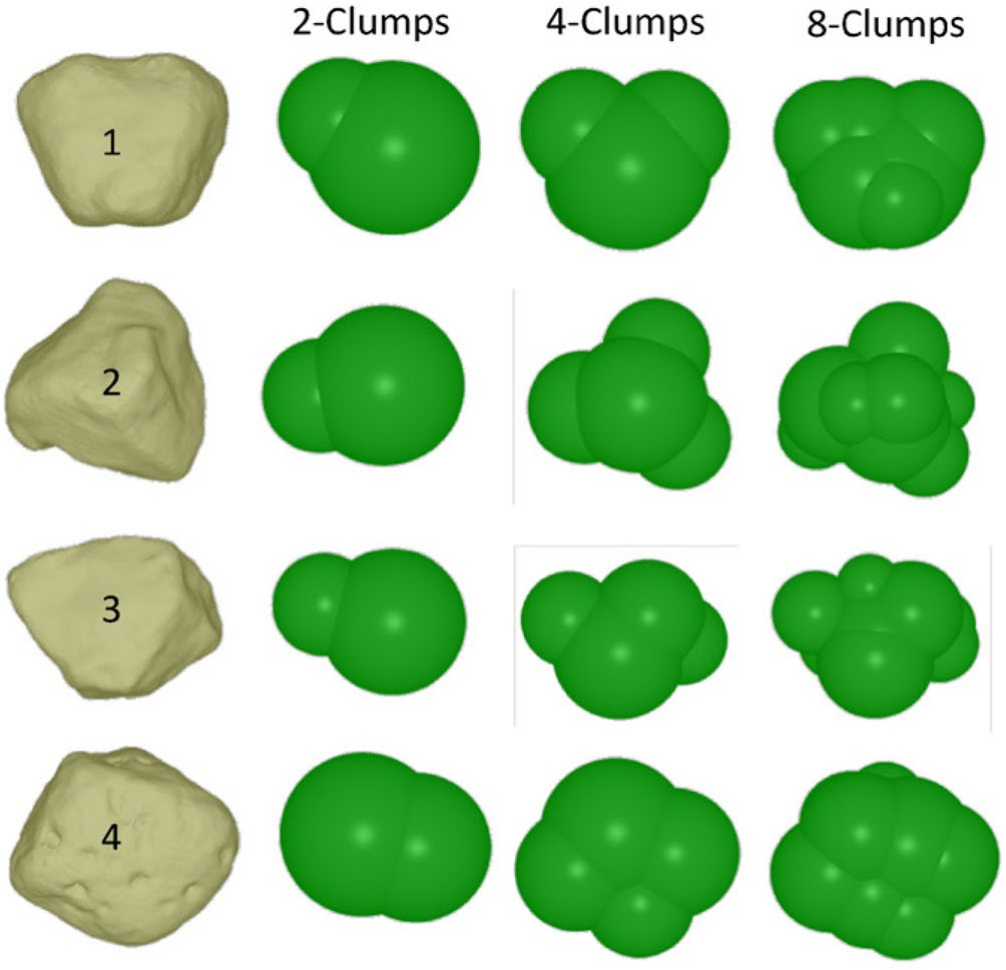
Scan maps and discrete element clumps^31^.

Several researchers emphasized the significance of the particle shape and generated geometric models using digital imaging techniques. The first step is to select several representative rock particles from a sample that is scanned for equivalent blocks, as shown in Fig. 6. Three sets of clusters were created using 2, 4, and 8 spheres, respectively, and are referred to as 2-cluster, 4-cluster, and 8-cluster. Generally, as the number of spheres increases, the differences in volume between the generated model and the actual block decrease. These are some modeling experiences from the perspective of shape.

The mechanical properties of different materials can be very different, so the calibration experience of different materials may also vary greatly. The calibration experience of some rock materials is discussed in this part. According to the experience of previous tests, one can determine the initial microscopic parameters as the initial values for updating. These initial parameters are determined based on experience, which can help reduce the computational cost of calibration in the trial-and-error method.

Based on experience, the Poisson’s ratio of rock is mainly influenced by the stiffness ratio (i.e., $pb\_K^{*}$). The main influencing factor of Young’s modulus is the contact modulus of the particles (i.e., $pb\_E^{*}$). For the failure model of the material, experience indicates that the ratio of tensile strength ${\sigma _{c} }$ and cohesion *c* controls the failure mode of the material. In uniaxial tests, when the ratio is larger, the specimen is more likely to show shear failure. When the ratio is smaller, the specimen is more likely to show brittle failure. According to Shi^82^ experience, this ratio is between 0.5 and 2. One can determine the ratio value based on the failure mode. After determining the ratio, the other tensile strength ${\sigma _{c} }$ can also be calculated by assuming a cohesive *c*. The two values obtained are called the reference bonding strength. Based on the reference bonding strength, one can multiply it by a coefficient (e.g., 0.5, 1.0, 2.0) to obtain different peak strengths.

#### Validation

**Figure 7:**
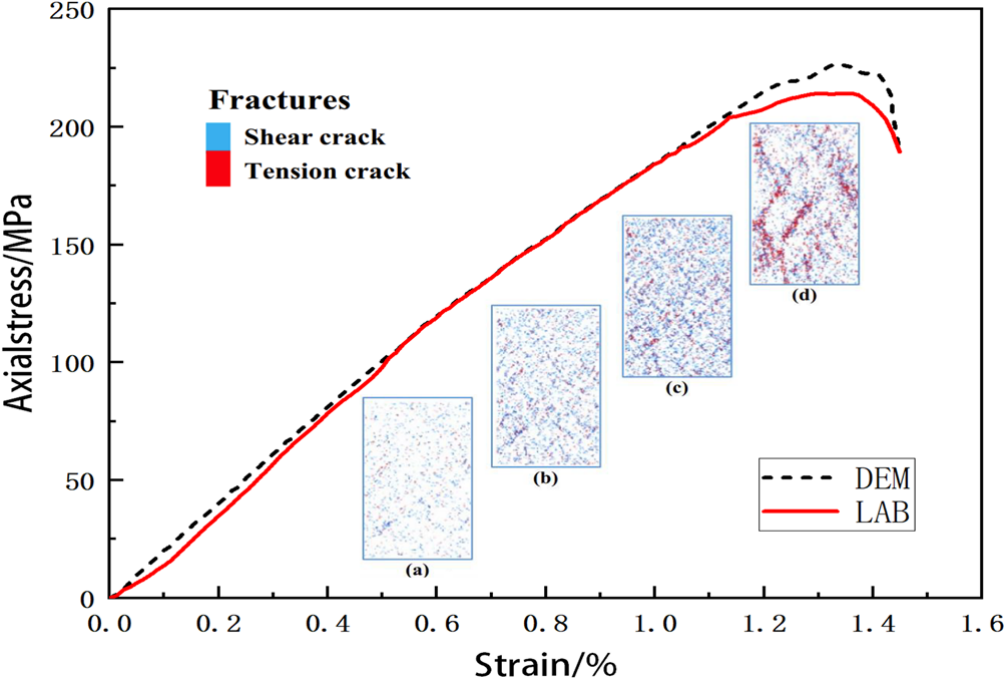
Comparison of experiment and simulation^82^.

Shi believes that DEM simulations not only require reasonable microscopic parameters but also need to consider the mineral distribution within the microscopic structure of rocks^82^. Therefore, he employed digital imaging techniques to characterize the microscopic structure for geometric modeling. This model was subsequently compared with experimental data, as illustrated in Fig. 7. The results demonstrate that DEM simulations exhibited marginally elevated stresses in the early phases of the experiment, which later matched closely with the experimental curve during the middle phase. However, the DEM simulation produced markedly higher stresses than experimental data during the onset of cracks. The calculated Young’s modulus from the DEM simulation is 20.5 GPa while that from the experiment is 21.04 GPa. The relative error between them is 2.57%. Note that the relative error is defined by $\left| E _{c}- E _{e}\right| /E_{e}$, where $E_{c}$ is Young’s modulus from DEM simulation while $E_{e}$ is Young’s modulus from the experiment. Moreover, the uniaxial compressive strength from the experiment is 225.4 MPa while that of the DEM simulation is 216.43 MPa. The relative error between them is 4.04% which demonstrates a similarity. The strain value of the peak point from the DEM simulated curve approximates that of the experimental curve. The DEM simulated data are in good agreement with the test results which validates the calibration method.

#### Discussion

Many researchers use their engineering experience to guide DEM calibration. These experiences help scholars complete calibration faster and more accurately. However, there are still some issues with empirical methods that cannot be ignored. A certain amount of trials are still required. Empirical methods may help scholars find the initial value or the direction of calibration faster, but it’s not a one-shot solution. Trial and error is required, and the calibration is still somewhat blind.

Based on these issues, some researchers have proposed the DOE method to carry out the calibration for quickly and scientifically determining the values of microscopic parameters, which may reduce the computational cost.

### Design of experiments method

DOE is a method of using experimental design to quickly identify the linear and nonlinear relationships between macroscopic and microscopic parameters. Based on some conditions, a set of optimization equations are developed. By solving these equations, the specific values of microscopic parameters can be obtained without further trial and error.

Yoon^40^ applied the DOE methodology to DEM parameter calibration. Then, numerous researchers have conducted extensive research on the DOE method. Kevin^92^ utilized an appropriate experimental design (Taguchi method) and concluded that individual parameter calibration was unsuitable. Yan and other scholars^93–97^performed calibration on different materials using the DOE method, explored the impact of microscopic parameters on macroscopic parameters and further proposed corresponding adjustment criteria. Deng Shuxin and other researchers^56,98–101^ used the DOE method to calibrate a 3D model and they discovered insignificant deviations in the results. Peng and other researchers^102–104^ believed that the Plackett-Burman (PB) design was insufficiently accurate and therefore developed various methods, such as spherical symmetry design and central composite design. These investigations have made significant contributions to DEM calibration. Nguyen^105^ simulated multiple DEM models of tensile tests with varying microscopic parameter values to generate a macroscopic parameter response database. They analyzed the database with thousands of data sets using nonlinear least squares and derived analytical laws.

#### Calibration principle

Firstly, macroscopic and microscopic parameters are selected. The macroscopic parameters of the homogeneous isotropic linear elastic material are taken such as the uniaxial compressive strength $\sigma _{u}$, Young’s modulus E, Poisson’s ratio $\upsilon$ and uniaxial tensile strength $\sigma _{t}$. The microscopic parameters of the linear contact bond model are determined such as the effective modulus $E^{*}$, stiffness ratio $K^{*}$, friction coefficient $\mu$, tensile strength $\sigma _{c}$ and shear strength $\tau _{c}$. The macroscopic and microscopic parameters are expressed by $\varvec{\alpha }$ and $\varvec{ \beta }$ respectively, as shown in equation (10) and equation (11).

10 $$\begin{aligned} \varvec{\alpha }= & {} \begin{bmatrix} \sigma _{u}&E&\upsilon&\sigma _{t} \end{bmatrix}^{T} \end{aligned}$$

11 $$\begin{aligned} \varvec{ \beta }= & {} \begin{bmatrix} E^{*}&K^{*}&\mu&\tau _{c}&\sigma _{c}&1 \end{bmatrix}^{T} \end{aligned}$$

Then several sets of numerical simulation experiments were performed. Different microscopic parameters were selected for each set of experiments and macroscopic parameters were calculated based on the simulation results.

After obtaining the simulation results, the functional relationship between the microscopic and macroscopic parameters is established as shown in equation (12).

12 $$\begin{aligned} \varvec{\alpha } =f(\varvec{\beta } ) \end{aligned}$$

More specifically, we establish a linear relationship.

13 $$\begin{aligned} \varvec{\alpha }=\textbf{A} \varvec{\beta } \end{aligned}$$

Where $\textbf{A}$ is a matrix of linear coefficients, denoted specifically as $$\textbf{A} =\begin{bmatrix} \textbf{X} _{1}&\textbf{X} _{2}&\textbf{X} _{3}&\textbf{X} _{4} \end{bmatrix}^{T}$$. $\textbf{X} _{1}$ is a vector of linear coefficients of ${\sigma _{u}}$. $\textbf{X} _{1}$ is denoted specifically as $$\textbf{X} _{1} =\begin{bmatrix} a_{1}&a_{2}&\dots&a_{6} \end{bmatrix}^{T}$$. The remaining parameters follow this rule similarly. According to equation (13), the key to establishing the relationship between macroscopic and microscopic parameters is to determine the coefficient matrix $\textbf{A}$. There are various ways to obtain the matrix A. Here, we can solve $\textbf{X} _{1}$, $\textbf{X} _{2}$, $\textbf{X} _{3}$ and $\textbf{X} _{4}$ respectively. Taking $\textbf{X} _{1}$ as an example, we first establish the following equation (14)

14 $$\begin{aligned} \sigma _{u}=\textbf{X} _{1}^{T}\varvec{\beta } \end{aligned}$$

There are 6 unknowns in $\textbf{X}_1$, and at least *n* equations (where $n\ge 6$) are required to solve equation (14). Therefore, it is necessary to conduct n sets of DEM simulations of the same system with varying microscopic parameters. Each set of simulations aims to determine the corresponding macroscopic parameters. Finally, the least squares method can be used to obtain $\textbf{X}_1$. By analogy, $\textbf{X}_2$, $\textbf{X}_3$, and $\textbf{X}_4$ can also be obtained, leading to the determination of *A*.

The previously obtained relationship between macroscopic response and microscopic parameters is linear. To further obtain the non-linear relationship between macroscopic response and microscopic parameters, two microscopic parameters with the most significant impact on a particular macroscopic parameter are selected. For instance, Yoon^40^ identified $\sigma _{c}$ and $\tau _{c}$ as the two microscopic parameters that have the greatest impact on tensile strength. Considering the mutual influence between these two coefficients, a non-linear relationship between macroscopic parameters and microscopic parameters is established according to equation (15).

15 $$\begin{aligned} \sigma _{t}=\varvec{x}_{1}^{T}\varvec{\gamma } \end{aligned}$$

Where $$\varvec{\gamma }=\begin{bmatrix} \tau _{c}&\sigma _{c}&\tau _{c}\sigma _{c}&\tau _{c}^{2}&\sigma _{c}^{2}&1 \end{bmatrix}^{T}$$ is the microscopic parameter vector and the nonlinear coefficient vector is denoted as $$\textbf{x} _{1}=\begin{bmatrix} e_{1}&e_{2}&\dots&e_{6} \end{bmatrix}^{T}$$. The coefficient vector $\textbf{x} _{1}$ in equation (15) can be obtained via the least square method.

To minimize the discrepancy between the numerical simulation results and the physical experiment results, an optimization problem is formulated to seek the optimal values of the microscopic parameters in DEM. The objective of the optimization is to minimize the absolute difference between the laboratory-measured material parameters and the DEM simulation parameters. The optimization problem can be formulated as shown in equation (16).

16 $$\begin{aligned} Minimize\left| Simulation \ result - Laboratory \ test \ result \right| \rightarrow 0. \end{aligned}$$

To achieve the optimization objective, it is necessary to impose linear equality and linear inequality constraints, i.e., the macroscopic parameters must satisfy linear and nonlinear equations. In addition to these constraints, other inequality conditions must also be satisfied. For instance, the ratio of uniaxial compressive strength to Brazilian tensile strength in the bond-particle model should be between 3 and 10, etc^106–109^.

According to the above analysis, the optimization problem has four objective functions, six output variables, and several constraints, which contain quadratic terms and belong to a multi-objective nonlinear constraint problem.

#### Validation

**Figure 8:**
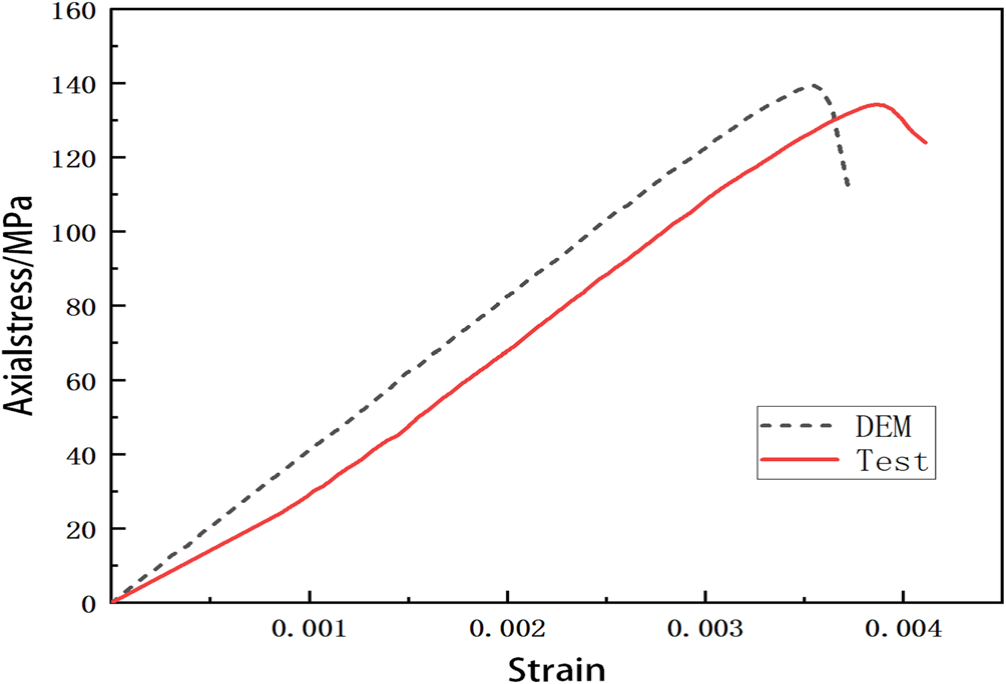
Comparison of experiment and simulation^104^.

The comparison between the stress-strain curves obtained from DEM simulation and experiments, as conducted by Li^104^, is shown in Fig. 8. Figure 8 shows that the DEM simulated curve maintains an ideal elastic response, characterized by a linear trajectory before reaching the peak. The experimental curve and the DEM simulated curve bifurcate slightly at the beginning of loading which may result from insufficient initial exposure between the experimental equipment and the test specimen. Then the experimental curve ascents approximately parallel to the DEM simulated curve that demonstrates comparable Young’s moduli. The calculated Young’s modulus from the DEM simulation is 41.07 GPa while that from the experiment is 40.17 GPa. The relative error between them is 2.24%. Note that the relative error is defined by $\left| E _{c}- E _{e}\right| /E_{e}$, where $E_{c}$ is Young’s modulus from DEM simulation while $E_{e}$ is Young’s modulus from the experiment.

Moreover, the uniaxial compressive strength from the experiment is 136.4 GPa while that of DEM simulation is 139.49 GPa. The relative error between them is 2.26% which demonstrates a similarity. However, the strain value of the peak point from the experimental curve is a little larger than that of the DEM simulated curve which may result from the bifurcations of curves at the beginning of loading.

Table 2 presents a comprehensive compilation of experimental and simulated data for various materials. The DEM simulated data are in good agreement with the test results which validates the calibration method.

**Table 2:** Simulated and experimental results of macroscopic mechanical parameters for different rocks under uniaxial compression^104^.

Rock type	*E*			V			$\sigma _{\textrm{u}}$		
	Cal./ GPa	Exp./ GPa	Error/%	Cal./GPa	Exp./GPa	Error/%	Cal./GPa	Exp./GPa	Error/%
Red sandstone	11.12	10.64	4.22	0.272	0.273	0.74	63.96	61.11	0.06
	10.67	10.67	0.28	0.27	0.27	1.11	64	64	4.52
Lac du Bonnet	69.08	68.91	0.12	0.26	0.262	0	206.4	199.71	3.20
granite	69	69	0.13	0.26	0.27	2.96	200	200	0.14
Carrara marble	50	49	2.04	0.228	0.23	0.87	94.44	101	6.50
BS granite	41.07	40.17	2.24	0.268	0.27	0.74	139.49	136.4	2.26
Wonju granite	47.31	46	2.85	0.216	0.22	1.82	165.71	173	4.21
Hwangdeung granite	51	50.7	2.04	0.278	0.28	0.87	94.44	162	6.50
Rock-like material	17.6	16.98	3.65	0.186	0.19	2.10	60	63.19	5.05

#### Discussion

Although the DOE method can obtain the sensitivity of each microscopic parameter to the macroscopic parameter in a relatively scientific, fast, and accurate manner, and can calculate the specific values of the required microscopic parameters, there are still some disadvantages that cannot be ignored. The calibration results of the design of experiments are highly sensitive to the selection of microscopic parameters. Inappropriate choices of microscopic parameters can lead to inaccurate calibration results in DOE. Furthermore, there may exist coupling relationships among the parameters, indicating that the adjustment of one parameter can affect others. Neglecting the coupling relationships between parameters during DOE calibration can result in inaccurate outcomes. The formula, calibrated by DOE, is specifically applicable to particular material models with different parameter ranges. When the microscopic parameters fall outside the defined range, the reliability of the calibration formula diminishes. Moreover, DOE exhibits limitations when it encounters nonlinear issues, which may introduce substantial errors.

With the development of computer technology, machine learning has become increasingly popular. The use of machine learning to analyze the nonlinear relationships between microscopic parameters and macroscopic parameters seems to be a further step beyond the DOE method.

### Machine learning method

Machine learning is a method of data analysis. In machine learning, algorithms are trained on data, that identify patterns and relationships in the data. These algorithms can then make predictions or take actions based on new data that they have not seen before. Compared to the design of experiments method, machine learning methods can more efficiently handle high-dimensional and nonlinear issues. Various machine learning methods have been utilized by researchers to address parameter calibration issues, including random forest method^44,56^, support vector machine method^44,56^, Bayesian filtering method^110–113^, and neural network method^30,42,61,95,114^.

The fundamental principle of random forest is to train data by constructing an ensemble of decision trees, each independently making predictions. The final prediction is obtained through collective decision-making and voting among these trees. This approach offers notable advantages, such as high accuracy and robustness, making it particularly suitable for handling high-dimensional and large-scale datasets. Shentu employed the random forest algorithm for the calibration of the discrete element method and observed promising precision. The calibration process involved the utilization of 500 data sets^44^. This method has the possibility of overfitting issues when a small amount of data is available. As a result, the model may exhibit high accuracy on the training data but struggle to deal with the new data. Support vector machine calibration is primarily employed to determine an optimal hyperplane for classification and prediction. The notable advantage of this method lies in its robustness when it encounters limited samples. Shentu et al. has applied the support vector machine algorithm for the calibration of the discrete element method with 500 data, observing favorable precision in calibration results^44^. However, this method is susceptible to noise and overlapping data. Bayesian filtering calibration primarily utilizes the probabilistic inference approach. Bayesian filtering calibration leverages prior experience as the initial estimate, and it is iteratively updated through comparisons with simulated experiments, resulting in refined posterior estimates. This iterative process continues until the desired convergence is achieved. The notable advantages of this method lie in its capacity to effectively handle uncertainty and noise, as well as its adaptability to dynamic system variations. Cheng et al. applied Bayesian filtering for the calibration of the discrete element method with 1000 instances and observed highly accurate predictions^110^. However, it demands a meticulous specification of prior experience. Neural networks are composed of interconnected artificial neurons, which are fundamental for receiving input signals, performing computations, and generating output signals. These neurons collectively form intricate connections within the neural network. Typically, a neural network comprises input layers, hidden layers, and output layers. The training process of a neural network involves iteratively adjusting the weights between neurons for high prediction accuracy. Neural networks can effectively model complicated nonlinear relationships and exhibit strong adaptive learning capabilities. Long et al. employed neural networks for parameter calibration, leveraging a dataset consisting of 270 observations, and the result demonstrated a substantial improvement of approximately 50% in predictive accuracy compared to the traditional design of experiments approach^114^.

In summary, random forest demonstrates high accuracy when it owns large-scale data. However, it is prone to overfitting when the data is limited. Conversely, support vector machines exhibit good precision when working with limited samples, but are susceptible to the influence of noise. Bayesian filtering proves effective in handling noise, but it requires prior experience for training. The neural networks effectively model complicated nonlinear relationships and exhibit strong adaptive learning capabilities.

#### Calibration principle

**Figure 9:**
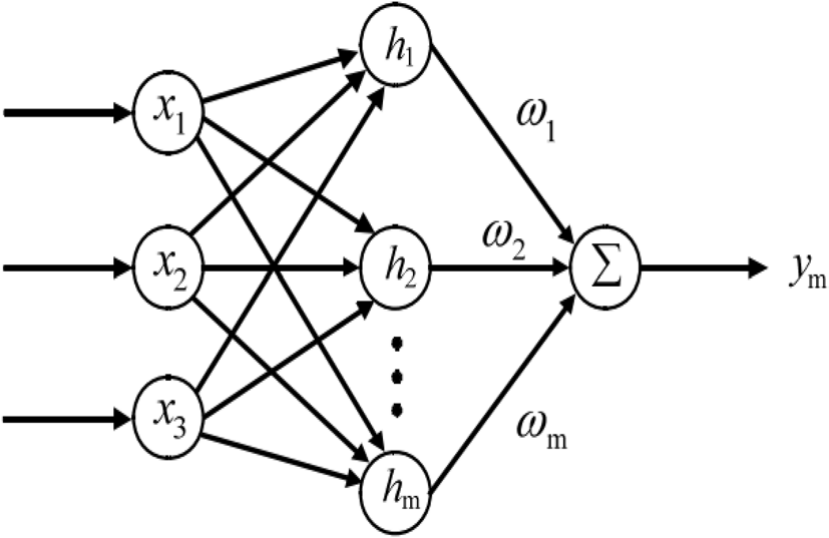
Schematic diagram of the machine learning^115^.

A neural network comprises three types of layers: an input layer, a hidden layer, and an output layer. The input layer receives external information, while the hidden layer facilitates the learning process within the network. Finally, the output layer generates the ultimate information. Each layer is composed of multiple neurons. The neurons in adjacent layers of the neural network are interconnected through synapses with associated weights. These weights act as parameters that regulate the significance or influence of the input signals transmitted between the layers (as depicted in Fig. 9). The weights play a pivotal role in shaping the network and they are adjusted through training the network using collected data.

The initial step in the parameter calibration via a neural network entails data collection. It is essential to ascertain the microscopic parameters with reasonable ranges. Upon establishing the microscopic parameters, several simulations should be conducted. The outcomes of each simulation, encompassing both microscopic and macroscopic parameters, are meticulously recorded to assemble a comprehensive dataset.

After data collection, data splitting is a crucial process that involves dividing the entire dataset into two distinct subsets: the training subset and the testing subset. The training subset is utilized to train the neural network, while the testing subset is employed to assess the performance of the trained neural network model by comparing its outputs with the target outputs.

Following the data splitting, the determination of the neural network model’s architecture becomes imperative, encompassing the configuration of the input layer, hidden layer(s), and output layer. As previously indicated, the input layer necessitates the inclusion of neurons, where the count should correspond to the number of microscopic parameters. Similarly, the number of neurons within the output layer should align with the count of macroscopic parameters. Both the input and output layers should derive data from the same set. Furthermore, in this study, a single hidden layer is exclusively employed.

After the establishment of the network architecture, the subsequent step involves the selection of a suitable training algorithm. Numerous training methods exist for neural networks, including the backpropagation algorithm, stochastic gradient descent algorithm, and various others. The judicious choice of an algorithm can significantly enhance computational efficiency.

Upon completing data collection, finalizing the neural network architecture and training algorithm, the neural network can be subjected to training data to ascertain the neural network model (e.g., the weights). This resulting neural network model can then be effectively employed for parameter calibration purposes.

#### Validation

Zhai used neural networks training for parameters calibration that chose the marble of Jinping Hydropower Station as the calibration object. The calibration results are shown in Table 3. From Table 3, one can find that the maximum error is 27.985% from cohesion strength while the minimum error is 1.527%^95^ from cohesion strength too. The maximum relative error for Young’s modulus is 21.176% while the maximum relative error for Poisson’s ratio is 9.615%. The maximum relative error for uniaxial compressive strength is 12.031%. These findings suggest that a larger dataset may be necessary for the parameters calibration with neural networks. Insufficient data during the calibration process can result in significant errors.

**Table 3:** Machine learning: comparison of experimental and simulated data^95^.

Property	Laboratory test results		Simulation results		Relative error	
Unconfined compressive strength (MPa)	77.3	77.3	68	80	12.031%	3.493%
Young’s modulus (GPa)	85	85	67	88	21.176%	3.529%
Poisson’s ratio	0.26	0.26	0.285	0.28	9.615%	7.692%
Cohesion strength (MPa)	26.8	22.9	19.3	22.55	27.985%	1.527%
Internal friction angle ($^{\circ }$)	27.1	33.4	30.549	31.791	12.726%	4.816%
Average error					16.714%	4.211%

#### Discussion

Although machine learning calibration has addressed nonlinear problems effectively, several issues cannot be overlooked. This includes the requirement for voluminous data and the time-consuming nature of manual operations, resulting in low efficiency. With insufficient data, results may be less reliable. Due to the disadvantages of machine learning, some scholars have turned their research toward evolutionary algorithms.

### Evolutionary algorithms method

Evolutionary algorithms possess the attributes of self-organization, self-adaptation, and self-learning, and are capable of efficiently addressing complicated problems. Two evolutionary algorithms are employed for calibration, i.e., the differential evolution and the particle swarm optimization. Both of these methods do not need data for training. They adaptively generate data during the calibration process. The differential evolution algorithm randomly generates many sets of microscopic parameters. Then it pinpoints some sets of microscopic parameters whose macroscopic parameters are close to the given macroscopic parameters. The differential evolution algorithm then generates new sets of microscopic parameters from the pinpointed parameters through differential mutation and crossover operations^47,116^. This process continues until the microscopic parameters meet the requirement. In particle swarm optimization algorithm^46,117^, each particle has a position and velocity and iteratively updates them based on information from individual and global best solutions.

Wang^46^ utilized the particle swarm optimization^117–120^ algorithm to automate the calibration of the microscopic parameters. The method is based on the concept of individual learning and evolution through mutual information sharing during the variation process, to achieve the target value. The method is automatically implemented, thereby avoiding the laborious and time-consuming manual operation, and showing considerable potential for practical applications. Ji^47,116^ proposed an optimized differential evolution calibration method that automatically calibrates the microscopic parameters to the target macroscopic parameters. Simulation experiments demonstrated that the macroscopic parameters, such as Young’s modulus, Poisson’s ratio, uniaxial compressive strength, and direct tensile strength, can be calibrated with a relative error of less than 5%. Furthermore, based on the calibration results, Ji conducted a uniqueness analysis of the microscopic parameters, unveiling the correlation between the microscopic parameters and the macroscopic mechanical behavior. These studies have made substantial contributions to the calibration.

#### Calibration principle

First, we assume the existence of a macroscopic parameter space whose dimensions are determined by the number of parameters in the macroscopic parameter vector $\varvec{\alpha }$. For example, if there are two parameters in $\varvec{\alpha }$, the macroscopic parameter space would be 2D. Each point in this space corresponds to a unique set of macroscopic parameters, which we refer to as the target value.

Next, m sets of microscopic parameter vectors $\varvec{\beta }$ are randomly selected (e.g., $\varvec{\beta }_1$, $\varvec{\beta }_2$, ..., $\varvec{\beta }_m$). The microscopic parameters in each $\varvec{\beta }$ vector can be related to a set of macroscopic parameters through numerical simulation experiments. In other words, each set of microscopic parameters corresponds to a point in the macroscopic parameter space. However, in general, the computed coordinates of these m microscopic parameters will not match the target value.

To address this issue, we need to update the microscopic parameter vectors $\varvec{\beta }$. Let the change of microscopic parameter vector $\bigtriangleup \varvec{\beta }_{i}$ be.

17 $$\begin{aligned} \bigtriangleup \varvec{\beta }_{i}=\begin{bmatrix} v_{1}&v_{2}&\cdots&v_{n} \end{bmatrix} \end{aligned}$$

Where $v_{i}$ is the change of per microscopic parameter. Since there is no evolutionary experience at the beginning, all these variables are random. The update is expressed in equation (18).

18 $$\begin{aligned} \textbf{Z} _{i}=\varvec{ \beta }_{i} +\bigtriangleup \varvec{\beta }_{i}=\begin{bmatrix} z_{1}&z_{2}&\cdots&z_{n} \end{bmatrix} \end{aligned}$$

At this stage, the coordinates are acquired by simulations. Equation (19) is used to determine whether the target is obtained.

19 $$\begin{aligned} min\left( \frac{\left| \sigma _{UCS}-\sigma _{u} \right| }{\sigma _{UCS} } ,\frac{\left| E_{ex}-E \right| }{E_{ex} } \right) \end{aligned}$$

Where $\sigma _{UCS}$ is the uniaxial compressive strength measured in the laboratory. $\sigma _{u}$ is the uniaxial compressive strength derived from numerical simulations. $E_{ex}$ is Young’s modulus measured in the laboratory. And *E* is Young’s modulus obtained from numerical simulations.

In fact, the smaller the value of equation (19), the closer the calculated value is to the target value. Based on equation (19), the optimal position location $\textbf{P}{i,pbest}$ for the evolution of $\varvec{Z}{i}$ and the optimal position $\textbf{P}_{gbest}$ for the evolution in these $\varvec{Z}$ can be determined.

20 $$\begin{aligned} \textbf{P} _{i,pbest}= & {} \begin{bmatrix} p_{p1}&p_{p2}&\cdots&p_{pn} \end{bmatrix} \end{aligned}$$

21 $$\begin{aligned} \textbf{P} _{gbest}= & {} \begin{bmatrix} p_{g1}&p_{g2}&\cdots&p_{gn} \end{bmatrix} \end{aligned}$$

Then, according to equation (20) and equation (21), the amount and position of all Z changes are updated again.

22 $$\begin{aligned} \bigtriangleup \varvec{\beta }_{i}^{k+1}= & {} w\bigtriangleup \varvec{\beta }_{i}^{k}+c_{1} r_{1}(\textbf{P} _{i,pbest}^{k}-\textbf{Z} _{i}^{k} )+c_{2} r_{2}(\textbf{P} _{gbest}-\textbf{Z} _{i}^{k} ) \end{aligned}$$

23 $$\begin{aligned} \textbf{Z} _{i}^{k+1}= & {} \textbf{Z} _{i}^{k}+\bigtriangleup \varvec{\beta }_{i}^{k+1} \end{aligned}$$

Where k is the number of iterations (starting from 1), w is the inertial learning factor, $c_{1}$ is the individual learning factor, $c_{2}$ is the social learning factor, and $r_{1}$ and $r_{2}$ are random numbers. w, specific values of $c_{1}$ and $c_{2}$, inappropriate numbers can lead to non-convergence, which is where the evolutionary algorithm is most prone to errors.

**Figure 10:**
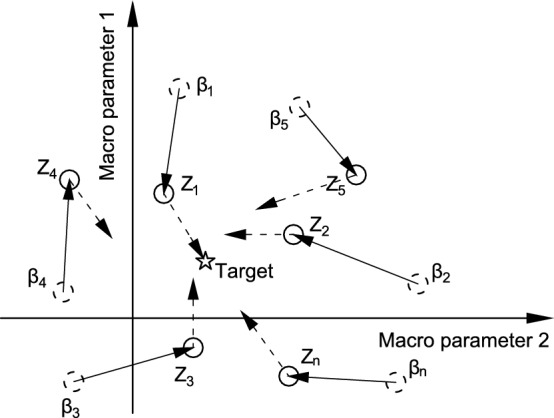
Schematic diagram of evolutionary process.

The termination condition for evolution is that the value of equation (19) is less than or equal to 0.05, and calibration will stop if the condition is met; otherwise, it will continue to iterate. The successful evolution diagram is presented in Fig. 10 below, where the stars marked with represent the position of the target value $\varvec{\alpha }$. The other solid circles are $\textbf{Z}$, the dashed drawn circles are $\varvec{\beta }$, and the solid arrows represent the position of the first move, and the dashed arrows represent the final position reached after several moves, which is also the process of evolution.

#### Validation

**Figure 11:**
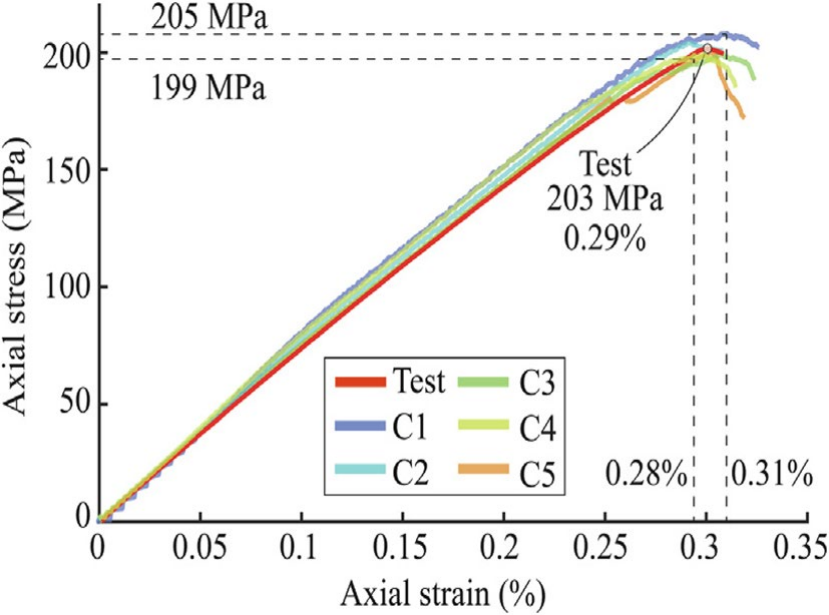
Evolutionary algorithm: comparison of experimental and simulated stress-strain^116^.

The calibrated model was compared with the experiment data, and Ji^116^ repeated the calibration process five times to verify the repeatability of the calibration, as shown in Fig. 11. One can observe a remarkable similarity in the slopes of the six stress-strain curves, suggesting a close approximation of Young’s modulus values. The annotated data on the graph indicates a minute relative error of 0.29% for the uniaxial compressive strength. These findings highlight a good calibration result, demonstrating a high fidelity of both the elastic and fracture stages of the material behavior.

#### Discussion

Evolutionary algorithms have made significant contributions to the computation of nonlinearity and automation. However, they still suffer from several limitations including a lack of dynamic adjustment, possibility to reach local optimum, resulting in low accuracy and difficulty in achieving convergence. Additionally, appropriate parameters must be chosen for different models to achieve optimal results.

### Theoretical derivation method

The DOE method, following the trial-and-error method, as well as the machine learning method and evolutionary algorithms method, utilize statistical methods in varying degrees. These mathematical algorithms provide researchers with the ability to quantify various phenomena and are seemingly a panacea for many complicated problems. They only offer an empirical approximation and lack precise descriptions of physical laws. Some researchers have pursued the calibration of DEM through theoretical derivations. Notably, Qu^45^ has made significant contributions to the theoretical derivation. This section primarily elucidates his theoretical idea about DEM calibration.

#### Calibration principle

The study focuses on a linear model and adopts the Voigt hypothesis^121^, which assumes uniform deformation throughout the domain. The basic idea for calibrating microscopic and macroscopic parameters is that the elastic energy density should be equal. In other words, the elastic energy density $\omega _{discrete}$ characterized by the DEM and the strain energy density $\omega _{continuum}$ described by classical continuum mechanics are equivalent, i.e., equation (24).

24 $$\begin{aligned} \omega _{discrete}=\omega _{continuum} \end{aligned}$$

In this context, the term elastic energy refers to the energy stored in a material as a result of deformation under contact forces^122^. The change of elastic energy occurs due to the displacement change on the tangential and normal contacts under the influence of external forces. Given that the topology of the particles does not change with their rotation^123^, the total stored elastic energy in the system is expressed by the following equation (25).

25 $$\begin{aligned} \Phi _{discrete}=\sum _{k=1}^{N_{c} }\Phi _{k}=\sum _{k=1}^{N_{c} }\left( \int _{0}^{ U_{k}^{n} }F_{n} d\delta _{n}+\int _{0}^{\ U_{k}^{s} }F_{s} d\delta _{s} \right) \end{aligned}$$

Where $\Phi _{discrete}$ is the elastic energy of all the domains. k represents the contact points of the particles. $\Phi _{k}$ is the strain energy of contact k. $N_{c}$ is the total number of contact points. $U_{k}^{n}$ and $U_{k}^{s}$ denote the normal and tangential relative displacements of contact k. $d\delta _{n}$ and $d\delta _{s}$ are the infinitesimal deformations in the normal and shear directions, respectively. $F_{n}$ and $F_{s}$ are contact forces in the normal and shear directions, i.e., $\left| F_{n} \right| =\left| F_{i,j}^{n} \right|$ and $\left| F_{s} \right| =\left| F_{i,j}^{s} \right|$.

The elastic energy density of the whole system is given by the following equation (26).

26 $$\begin{aligned} \omega _{discrete} =\frac{\Phi _{discrete} }{V} \end{aligned}$$

Where V is the total volume.

According to the theory of elasticity, the stress tensor $\sigma _{pq}$ of a continuum can be obtained by differentiating the elastic energy density with respect to the corresponding strain tensor $\varepsilon _{pq}$ as following equation (27).

27 $$\begin{aligned} \sigma _{pq}=\frac{{\partial \omega _{continuum} }}{\partial \varepsilon _{pq} }=\frac{\partial \omega _{discrete} }{\partial \varepsilon _{pq} } \end{aligned}$$

The elastic stiffness tensor can be obtained by differentiating the stress tensor $\sigma _{pq}$ with respect to the corresponding strain tensor $\varepsilon _{pq}$ as equation (28).

28 $$\begin{aligned} C_{pqmn}=\frac{\partial \sigma _{pq} }{\partial \varepsilon _{mn} } \end{aligned}$$

Equation (28) is a generalized description and can be applied to any particle packing. Under the condition that all particles are of equal size and have the same material properties, Equation (28) can be deduced as equation (29).

29 $$\begin{aligned} C_{pqmn}=\frac{2N_{c}r^{2}(2K_{i,j}^{n}+3K_{i,j}^{s} ) }{15V}(\delta _{pn}\delta _{qm}+\delta _{pm}\delta _{qn})+\frac{4N_{c}r^{2}(K_{i,j}^{n}-K_{i,j}^{s} ) }{15V}\delta _{pq}\delta _{mn} \end{aligned}$$

Where r is the particle radius. $\delta _{mn}$ is the Kronecker function.

Although the particle assembly is essentially amorphous and generally heterogeneous, the mechanical behavior of the particle assembly can still be considered as an elastomer in the case of small deformations. Therefore, based on the equation established by solid mechanics, the relationship between the equivalent elastic parameters and the particle scale parameters can be established. The elastic stiffness tensor $C_{pqmn}$ of the isotropic elastic solid from classical continuum mechanics is shown as following equation (30).

30 $$\begin{aligned} C_{pqmn}=\frac{E}{2(1+\upsilon )}(\delta _{pn}\delta _{qm}+\delta _{pm}\delta _{qn} )+\frac{E\upsilon }{(1+\upsilon )(1-2\upsilon )}\delta _{pq}\delta _{mn} \end{aligned}$$

Comparing equation (31) and equation (32), we get.

31 $$\begin{aligned} K_{i,j}^{n}= & {} \frac{3EV}{4N_{c}r^{2}(1-2\upsilon ) } \end{aligned}$$

32 $$\begin{aligned} K_{i,j}^{s}= & {} \frac{3EV(1-4\upsilon )}{4N_{c}r^{2}(1-\upsilon -2\upsilon ^{2} ) } \end{aligned}$$

**Figure 12:**
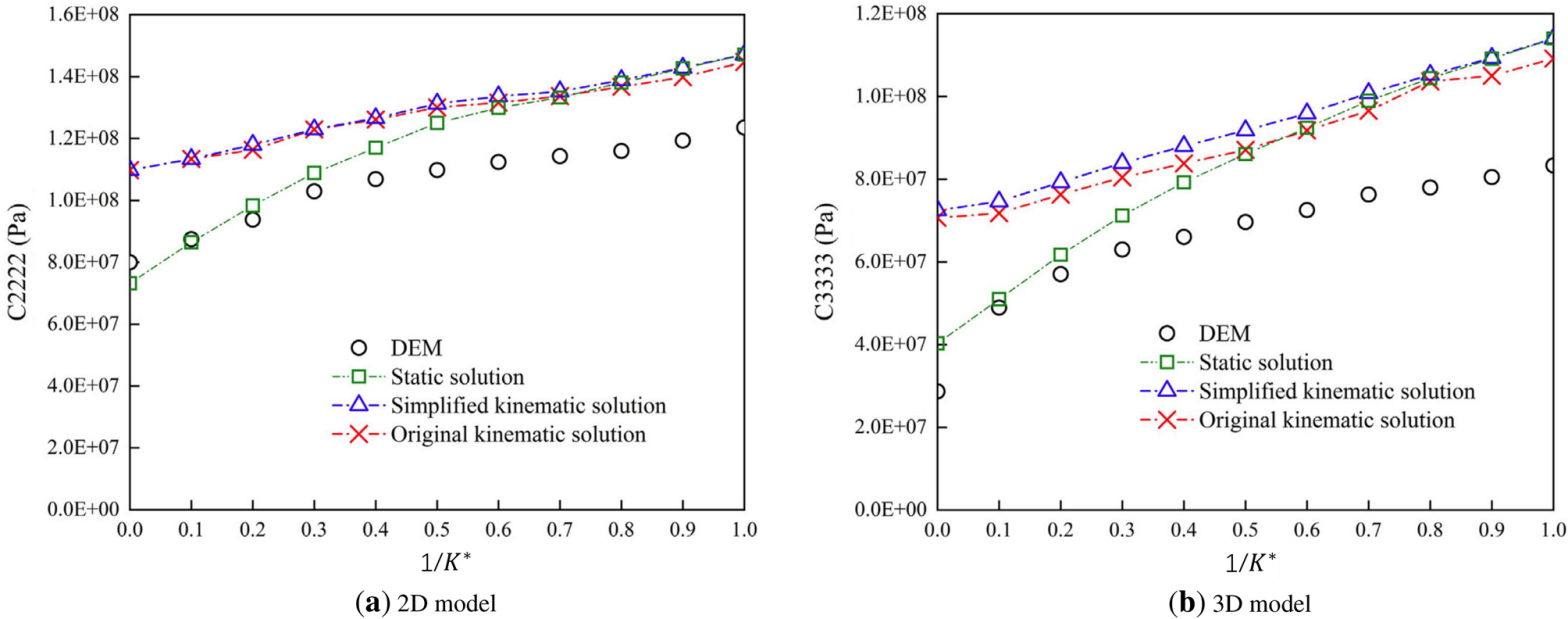
Components of equivalent stiffness tensor in DEM models with varied $1/K^{*}$^45^.

#### Validation and discussion

Qu conducted a comparative validation of the derived formulas. He used macroscopic parameters to obtain microscopic parameters via Eqs. (31) and (32). Then, he conducted simulations with the calculated microscopic parameters and obtained the simulated results, which are the macroscopic parameters. As illustrated in Fig. 12, the curves indicate a significant discrepancy between the simulated results and the analytical solutions (kinematic and static solutions) for different stiffness ratios. Generally, the simulated results should be between the kinematic and static solutions. From Fig. 12, we can see that the simulated results are much smaller than the analytical results. The discrepancy may be attributed to the non-uniform properties and arrangement of particles, which contradicts the assumption of homogeneity. The significant difference observed is attributed to the distinct modes of load transfer between particulate materials and continuous media.

## The applicability of calibrated parameters

Is it possible to directly apply parameters calibrated by other researchers? This question arises from the consideration that, apart from the microscopic parameters, additional factors such as particle distribution and porosity can potentially influence the macroscopic response. The primary aim of this section is to delve into the issue of parameter applicability, specifically exploring the circumstances under which calibrated parameters obtained by others can be reliably utilized.

**Figure 13:**
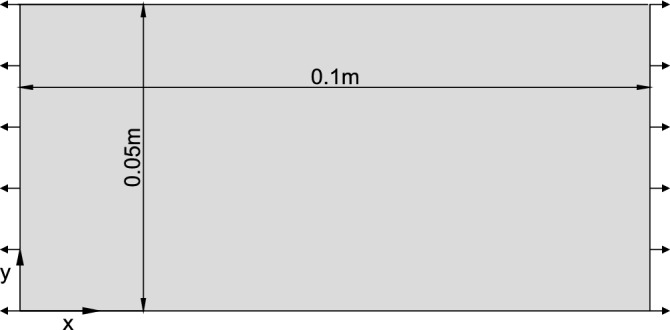
The geometry and boundary conditions.

### Influence of particle distribution

The DEM eschews the notion of a grid and instead relies on the spatial distribution of the particles. A fundamental phenomenon is found that simulation results are different when subject to identical microscopic parameters, different particle distributions, and particle size. This section explores the influence of particle distribution. Note that particle distribution refers to the physical arrangement or spatial distribution of particles within a given space.

**Table 4:** Microscopic parameters of different methods.

	$E^{*}$ (GPa)	$K^{*}$	$\mu$	$pb\_E^{*}$ (GPa)	$pb\_K^{*}$	$\sigma _{c}$(MPa)	*c*(MPa)
EM	2.8	1.45	0.6	2.8	1.45	20	20
DOE	22.63	1.379	0.5	22.63	1.379	150.91	150.91
ML	3.2	1	0.65	3.2	2.15	34.7	9.746
EA	1.76	0.57	6.28	55.96	2.94	64.04	73.63

#### Testing samples

To investigate particle distribution, this study employs four geometric models. The length of all the geometric models is 0.1 m and the width is 0.05 m. All four geometric models are subjected to the same uniaxial tension, achieved by specifying the velocity of the loading particles (shown in Fig. 13). Meanwhile, the four geometric models have the same porosity which is 0.16. The particles from four geometric models are randomly generated where radii ranging from 0.02 mm to 0.032 mm satisfy the Gaussian distribution. The main difference between the four models is the spatial distribution of the particles that are generated via the Gaussian random distribution function.

**Table 5:** Results of simulations with empirical parameters.

	Youngs modulus(GPa)	Poisson’s ratio
Data from paper	4.2	0.2
Model 1 data	3.38	0.135
Model 2 data	3.38	0.133
Model 3 data	3.38	0.135
Model 4 data	3.38	0.133

For the convenience of icon identification later, the empirical method, machine learning, and evolutionary algorithms are abbreviated as EM, ML, and EA respectively. Table 4 displays the microscopic parameters chosen from the papers among the various methods(EM data from Bahaaddini’s model^124^, DOE data from Peng’s model^102^, ML data from Guo’s model^30^, EA data from Wang’s model^46^).

#### Testing results

We calculate the macroscopic parameters from simulation results, which are shown in Tables  5, 6, 7, 8. Table 5 shows macroscopic parameters from Bahaaddini’s result and the four sets of geometric models. Note that all the microscopic parameters in geometric models are from Bahaaddini’s paper. Table 6 shows macroscopic parameters from Peng’s result and the four sets of geometric models. Note that all the microscopic parameters are from Peng’s paper. Table 7 shows macroscopic parameters from Guo’s result and the four sets of geometric models. Note that all the microscopic parameters are from Guo’s paper. Table 8 shows macroscopic parameters from Wang’s result and the four sets of geometric models. Note that all the microscopic parameters are from Wang’s paper.

**Table 6:** Results of simulations with DOE parameters.

	Youngs modulus (GPa)	Poisson’s ratio
Data from paper	20.21	0.2037
Model 1 data	27.8	0.129
Model 2 data	27.8	0.129
Model 3 data	27.7	0.129
Model 4 data	27.8	0.129

**Table 7:** Results of simulations with machine learning parameters.

	Youngs modulus (GPa)	Poisson’s ratio
Data from paper	3.62	0.244
Model 1 data	3.36	0.175
Model 2 data	3.37	0.173
Model 3 data	3.36	0.176
Model 4 data	3.37	0.173

**Table 8:** Results of simulations with evolutionary algorithms parameters.

	Youngs modulus (GPa)	Poisson’s ratio
Data from paper	23.3	0.17
Model 1 data	43.0	0.189
Model 2 data	43.0	0.188
Model 3 data	42.9	0.189
Model 4 data	43.0	0.188

**Figure 14:**
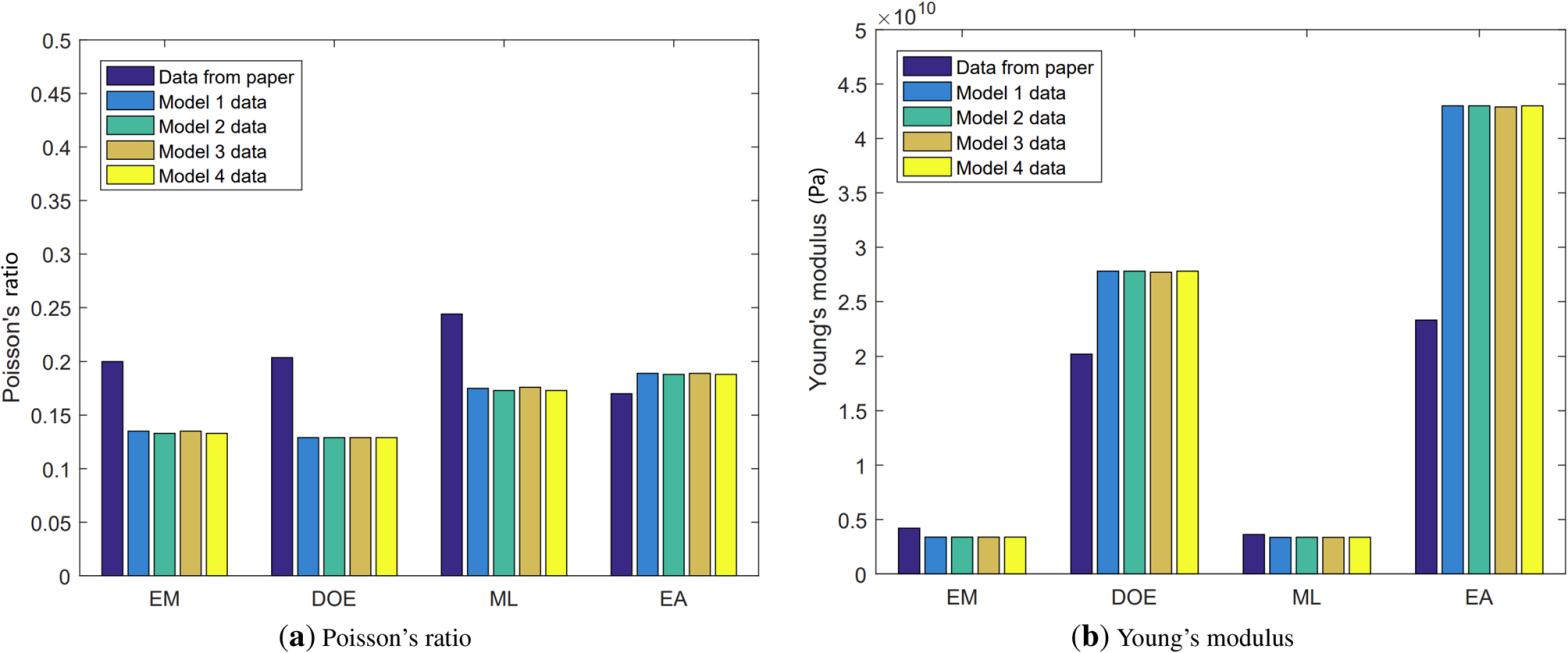
Comparison of results from different models.

To show the differences more visually, the above tables are formed into bar graphs of Poisson’s ratio and Young’s modulus. The results are shown in Fig. 14 as follows

#### Analysis of Poisson’s ratio

The statistical analysis of Poisson’s ratio is shown in Table 9 below. $v_{Max}$ is the maximum value of the Poisson’s ratio in the geometric models. $v_{Min}$ is the minimum value of the Poisson’s ratio in the geometric models. $v_{a}$ is the Poisson’s ratio in these papers. $\chi _{v1}$ is the relative error between the maximum and minimum values of the Poisson’s ratio in the geometric models. $\chi _{v2}$ is the maximum relative error between the Poisson’s ratio in the paper and the Poisson’s ratio in the geometric models.

In the EM group, the relative error between the maximum and minimum values of the Poisson’s ratio in the geometric models is 0.15%. 0.15% is calculated from $\left| (\upsilon _{Max}- \upsilon _{Min})\right| /{\upsilon _{Max}}$, and the following calculations are done by analogy. This indicates that the difference in Poisson’s ratio among the EM group is very small. Fig. 14 also shows that the Poisson’s ratio difference among the four geometric models generated by particles’ random distribution in the EM group is indeed relatively small. However, there is a large discrepancy between the data from the four geometric models and the data from the paper, with a relative error of 33.5% between the Poisson’s ratio in the paper and the minimum Poisson’s ratio in the geometric models.

In the DOE group, the relative error between the maximum and minimum values of the Poisson’s ratio in the geometric models is 0, indicating almost no difference. Figure 14 also shows that each group’s data in the DOE group are identical. However, there is still a large discrepancy between the data from the geometric models and the data from the paper, with a relative error of 36.67% between the Poisson’s ratio in the paper and the minimum Poisson’s ratio in the geometric model.

In the ML group, the relative error between the maximum and minimum values of the Poisson’s ratio in the geometric models is 1.73%, which indicates that the difference in Poisson’s ratio data among the four groups is also relatively small. Figure 14 also shows that the Poisson’s ratio difference among the four geometric models in the ML group is indeed relatively small. However, there is still a large discrepancy between the data from the geometric models and the data from the paper, with a relative error of 29.1% between the Poisson’s ratio in the paper and the minimum Poisson’s ratio in the geometric model.

**Table 9:** Poisson’s ratio from simulation results.

	$\upsilon _{Max}$	$\upsilon _{Min}$	$\upsilon _{a}$	${\chi }_{\upsilon 1}$	${\chi }_{\upsilon 2}$
EM	0.135	0.133	0.2	0.15%	33.5%
DOE	0.129	0.129	0.2037	0	36.67%
ML	0.176	0.173	0.244	1.73%	29.1%
EA	0.189	0.188	0.17	0.51%	10.05%

In the EA group, the relative error between the maximum and minimum values of the Poisson’s ratio in the geometric models is 0.512%, indicating that the difference in Poisson’s ratio data among the four groups is also relatively small. Figure 14 also shows that the Poisson’s ratio difference among the four geometric models in the EA group is indeed relatively small. However, there is still a large discrepancy between the data from the geometric models and the data from the paper, with a relative error of 10.05% between the Poisson’s ratio in the paper and the maximum Poisson’s ratio in the geometric models.

#### Analysis of Young’s modulus

The Young’s modulus group analysis calculation table is shown in Table 10 below. $E_{Max}$ is the maximum value of Young’s modulus in the geometric models. $E_{Min}$ is the minimum value of Young’s modulus in the geometric models. $E_{a}$ is Young’s modulus in these papers. $\chi _{E1}$ is the relative error between the maximum and minimum values of Young’s modulus in the geometric models. $\chi _{E2}$ is the maximum relative error between Young’s modulus in the paper and Young’s modulus in the geometric models.

In the EM group, the relative error between the maximum and minimum values of Young’s modulus in the geometric models is 0, with almost no difference. This indicates that the differences in Young’s modulus within the EM group are small, and it can be seen from Fig. 14 that each data calculation within the EM group is the same. However, there is a significant difference between the data from the four geometric models and the data in the paper. The relative error between Young’s modulus in the paper and the minimum Young’s modulus in the geometric models is 19.52%.

In the DOE group, the relative error between the maximum and minimum values of Young’s modulus in the geometric model is 0.36%, indicating a small difference. It can also be seen from Fig. 14 that there is little difference between each data calculation within the DOE group. However, there is still a significant difference between the data of the geometric model and the data in the paper. The relative error between Young’s modulus in the paper and the minimum Young’s modulus in the geometric models is 27.3%.

**Table 10:** Young’s modulus from simulation results.

	$E_{Max}\mathrm {(GPa)}$	$E_{Min}$ (GPa)	${E}_{a}$	${\chi }_{E1}$	${\chi }_{E2}$
EM	3.38	3.38	4.2	0%	19.52%
DOE	27.8	27.7	20.21	0.36%	27.3%
ML	3.37	3.36	3.62	0.29%	7.18%
EA	43.0	42.9	23.3	0.23%	45.8%

In the ML group, the relative error between the maximum and minimum values of Young’s modulus in the geometric models is 0.29%, and the differences in Young’s modulus among these four groups are small. It can also be seen from Fig. 14 that the differences in Young’s modulus among the four geometric models in the ML group are indeed small. However, there is still some difference between the data of the geometric model and the data in the paper. The relative error between Young’s modulus in the paper and the minimum Young’s modulus in the geometric model is 7.18%.

In the EA group, the relative error between the maximum and minimum values of Young’s modulus in the geometric model is 0.23%, and the differences in Young’s modulus among these four groups are also small. It can also be seen from Fig. 14 that the differences in Young’s modulus among the four geometric models in the EA group are indeed small. However, there is still a significant difference between the data of the geometric models and the data in the paper. The relative error between Young’s modulus in the paper and the maximum Young’s modulus in the geometric models is 45.8%.

#### Discussion

Based on the data presented above, it is apparent that the discrepancies in Poisson’s ratio and Young’s modulus among the four sets of randomly generated models are relatively small. However, in comparison to reported data^30,46,102,124^, these differences are quite significant. One can attribute this disparity to the lack of certain critical information, such as maximum and minimum particle radii and porosity. Notably, the four sets of random models are constructed with identical maximum and minimum particle radii and porosity settings, with the sole distinguishing factor being their spatial distribution of particles. The macroscopic properties from simulations exhibited minor differences. One can conclude that the influence of the spatial distribution of particles on macroscopic properties is negligible under the constraint of equivalent maximum and minimum particle radii and porosity.

### Influence of particle size

After studying the spatial distribution of the particles, this subsection focuses on the influence of the particle size. In this subsection, we generate three geometric models (i.e., model A, model B, and model C) with different features (i.e., dimension of geometric model and particle size). Then, we conduct simulations with consistent microscopic parameters to study the influence of these features on the macroscopic parameters.

**Table 11:** Results of simulations about Young’s modulus and Poisson’s ratio.

	Youngs modulus (GPa)	Poisson’s ratio
Model A	16.87	0.163
Model B	16.54	0.152
Model C	16.96	0.164

#### Testing samples

In order to investigate the influence of particle size in DEM, this study employs uniaxial tension tests with three sets of geometric models (see Fig. 13). The length of model A is 0.2 m and the width is 0.1 m. The length of model B is 0.1 m and the width is 0.05 m. The dimension of model C is the same as that of model B. These models are designed to maintain a consistent porosity of 0.18 and consistent microscopic parameters. Model A and Model B set identical maximum and minimum particle radii with values of 0.032 mm and 0.02 mm, respectively. Model C sets exhibited halved maximum and minimum radii. The linear parallel bond model is used in the simulations. The microscopic parameters are $E^{*}=15 \ GPa$, $K^{*}=1.8$, $\mu =0.5$, $pb_E^{*}=15 \ GPa$, $pb_K^{*}=1.8$, $\sigma _{c}=73.63 \ MPa$, $c=64.04 \ MPa$. The leftmost and rightmost particles are used as loading boundaries, and uniaxial tension is achieved by specifying the velocity of the loading particles during simulations.

#### Testing results

After we conducted the simulations, one can calculate the macroscopic parameters from simulation results, which are shown in Table 11.

#### Analysis of results

From the Table 11, Young’s modulus of model A and model B exhibit a relative error of 1.96% while that of model B and model C have the difference with a relative error of 2.47%. Model A and model C have a difference in Young’s modulus with a relative error of 0.53%, suggesting that their data are almost identical. The Poisson’s ratio of model A and model B exhibit a relative error of 6.75%. The Poisson’s ratio of model B and model C exhibit a relative error of 7.31%. Meanwhile, the relative error of Poisson’s ratio between model A and model C is only 0.6%, suggesting that their data are almost identical.

We propose the concept of size ratio, which aims to delve deeper into the influence specifically exerted by particle size. The size ratio is defined as follows,

33 $$\begin{aligned} \kappa =\frac{r}{r_{max} } \end{aligned}$$

where r is the equivalent radius and $r=\sqrt{\frac{A_{1}}{\pi } }$. $A_{1}$ is the area of the geometric model. $r_{max}$ is the radius of the maximum particle. Note that DEM has a high computational cost. To address this issue, the coarse-graining approaches are used, summarizing particles of the original system into grains. Lumping a pre-set number of particles into a spherical grain is the main principle of coarse-graining. The physical properties of the coarse-graining and the bigger-size of particles are different. The goal of the coarse-graining is to reduce computational cost while the goal of size ratio is to ensure the applicability of microscopic parameters ^36–38,125–131^.

The size ratios of each model are calculated (see Table 12).

**Table 12:** The different between the three models.

	Size ratio	Maximum particle (mm)	Length of model (m)	Width of model (m)
Model A	1.406	0.032	0.2	0.1
Model B	0.703	0.032	0.1	0.05
Model C	1.406	0.016	0.1	0.05

From Table 12, firstly we know the differences between model A and model B are the size ratio, length, and width of the model. They have the same maximum particle radius. Secondly, we know the differences between model B and model C are the size ratio and maximum particle. They have the same dimension as the geometric model. Finally, we know the differences between model A and model C are the maximum particle radius, length, and width of the model. They have the same size ratio.

According to Table 12, it can be seen that model A and model B have the same maximum particle radius and different dimensions, resulting in different size ratios. From Table 11, it can be observed that model A and model B have a relative error of 6.75% in Poisson’s ratio and a relative error of 1.96% in Young’s modulus. This suggests that the size ratio and dimensions may be factors affecting the macroscopic parameters of the models. Furthermore, based on Table 12, model B and model C have the same dimensions and different maximum particle sizes, resulting in different size ratios. From Table 11, it can be observed that model B and model C have a relative error of 7.31% in Poisson’s ratio and a relative error of 2.47% in Young’s modulus. This also indicates that the size ratio and maximum particle radius may be factors influencing the macroscopic parameters of the models. Moreover, according to Table 12, model A and model C have different maximum particle radii, different dimensions, and the same size ratio. Based on Table 11, it can be observed that model B and model C have a minimum relative error of 0.6% in Poisson’s ratio and a minimum relative error of 0.53% in Young’s modulus. This phenomenon rules out the individual influence of the maximum particle radius or size on the macroscopic parameters. In fact, it is the combined effect of the maximum particle radius and size (i.e., size ratio) that impacts the macroscopic parameters.

The relative errors of Young’s modulus and Poisson’s ratio for models A and C are 0.53% and 0.6% respectively, and one can find that the ratio of the size ratio of model A to that of model C is 1. When this ratio becomes 2 (i.e., the ratio of the size ratio of model A to that of model B), the relative errors of their Young’s modulus and Poisson’s ratio become 1.96% and 6.75%, respectively. We observe that when the size ratio doubles, the relative errors of Young’s modulus and Poisson’s ratio increase by nearly 4 times and 11 times, respectively. This indicates that even small variations in the size ratio lead to a significant change in the relative errors of the macroscopic parameters.

#### Discussion

Based on the above analysis, it can be concluded that the size ratio has an impact on the macroscopic parameters. Therefore, in order to apply the calibrated microscopic parameters, we need to ensure that the size ratio in the application simulation is consistent with the size ratio in the calibration simulation.

## Conclusions

One can find that the discrete element method provides a route for studying mechanical response including elastic deformation and structure failure. This study describes the governing equations of the discrete element method and pinpoints the microscopic parameters involved in different constitutive models. Since it has difficulties to experimentally measure the microscopic parameters, researchers proposed to obtain them via calibration strategies that were described in detail.

The calibration method initially applied by scholars is the trial-and-error method. It is very easy to operate. However, it has high computational costs. Furthermore, the trial-and-error method lacks scientific rigor, and calibrated parameters may only map into a subset of the macroscopic parameter space close to the true solution. The empirical method helps scholars find a reasonable initial value or the direction of calibration which highly reduces the computational cost. However, a certain amount of trials are still required. It’s not a one-shot solution. The DOE method studies the sensitivity of each microscopic parameter to the macroscopic parameter and constructs formulas for macroscopic parameters and microscopic parameters. The formulas can calculate the specific values of the required microscopic parameters. However, the formula is specifically applicable to particular material models with different parameter ranges. Moreover, DOE has limitations in dealing with nonlinear issues. Machine learning calibration has addressed nonlinear problems effectively. However, it requires voluminous data. With insufficient data, results may be less reliable. The evolutionary algorithm for parameter calibration does not require a training process and it has made significant contributions to the calculation of nonlinearity. The evolutionary algorithm has high stability, and the calibrated results are compared with the experimental values, with a relative error of only 0.29%. The theoretical derivation method directly derives the formulas between macroscopic and microscopic parameters via assumptions. However, the assumption conditions are strict, so the theoretical values differ greatly from the experimental results. These mentioned calibration methods have made great contributions to the calibration of the discrete element method. Among them, the evolutionary algorithm is a promising calibration method with high accuracy.

Moreover, the applicability of calibrated parameters is an additional concern because particle distribution and size may influence the mechanical response of structures under loading. After simulations, we find that (1) during the elastic phase, the spatial distribution of particles has little influence on the simulation results when the porosity, maximum radius, and minimum radius of particles are identical in both the geometric calibration model and that for applications. Simulation results uncover that the maximum relative error of Young’s moduli is 0.36%, while that of Poisson’s ratios is 1.73%. (2) We define the concept of size ratio. Then we find that the size ratio has a great influence on the simulation results. To ensure the applicability of calibrated parameters, the size ratio in different geometric models should be identical. This study identifies the application conditions of the calibrated parameters.

## Data Availability

The datasets used and/or analyzed during the current study are available from the corresponding author on reasonable request.
